# Relationship between polymorphisms and mutations at rs7125942 and rs3736228 of LRP5 gene and bone metabolism in postmenopausal women

**DOI:** 10.1186/s13018-024-04579-1

**Published:** 2024-02-01

**Authors:** Jun Li, Ya Li, Siyuan Li, Yunqiu Lu, Partab Rai

**Affiliations:** 1 Department of Endocrinology and Metabolism, The First Affiliated Hospital, Shihezi University, Shihezi, 832002 Xinjiang China; 2 Medical School of Shihezi University, Shihezi, 832002 China; 3 Ocaf Hospital, Karachi, Pakistan

**Keywords:** Gene polymorphisms, LRP5, Osteoporosis, T2DM

## Abstract

**Objective:**

To analyze the relationship between the polymorphism and mutation of rs7125942 and rs3736228 locus in the low-density lipoprotein receptor-related protein 5 (LRP5) genotype and bone mineral density (BMD) in postmenopausal women in Xinjiang, China, to provide a basis for prevention and treatment of the disease.

**Methods:**

According to the results of dual-energy X-ray (DEXA) determination of BMD, the 136 subjects were divided into three groups: Group A: normal bone mass, Group B: osteopenia, Group C: osteoporosis. 1. Age, body, mass index (BMI), and menopause of all subjects were recorded. 2. Fasting blood glucose (FBG), glycosylated hemoglobin (HbA1c), calcium (Ca), phosphorus (P), alkaline phosphatase (ALP), and clinical biochemical data were determined. 3. LRP5 locus polymorphisms were determined by time-of-flight mass spectrometry.

**Results:**

1. Compared with group A, the age, ALP, Cr, and BUN levels in group B and group C were increased, but UA levels were lower (*P* < 0.05), and Serum P was higher in the group C (*P* < 0.05). 2. There was no statistically significant difference in the prevalence of diabetes between the three groups (*P* > 0.05). 3. The ROC curves for different BMD sites such as L1, L2, L3, L4, L total, and femoral neck were 0.929, 0.955, 0.901, 0.914, 0.885, and 0.873 (*P* < 0.01). 4. At rs7125942 locus, there was statistically significant difference in the distribution of wild-type (CC) and mutant (CG) with the normal bone mass (NBM) group and the abnormal bone mass (ABM) group (*P* < 0.05). 5. At rs7125942 locus, compared with wild-type (CC), mutant (CG) had lower LDL and FPG in NBM group (*P* < 0.05), and lower serum ALP in the ABM group (*P* < 0.05). At rs3736228 locus, the BMD (Femoral neck) of mutant (CT/TT) was lower than that of wild-type (CC) in the NBM group (*P* < 0.05). 6. Age and menopausal years were negatively correlated with BMD of the femoral neck and L1-4 (*P* < 0.05), and BMI and TG were positively (*P* < 0.05), and the results of multiple linear regression analysis showed that age, BMI, and TG were both independent factors affecting BMD (*P* < 0.05).

## Introduction

Low bone mass and micro-architectural disturbance of bone tissue are characteristics of osteoporosis, a systemic metabolic illness that increases bone fragility and fracture susceptibility [1]. Osteoporosis (OP) disproportionately affects elderly and postmenopausal women. Twenty-five percent of patients over 50 who suffer an osteoporotic hip fracture are predicted to pass away within a year [2]. A total of 200 million people globally are at danger for osteoporosis-related fractures, which can increase morbidity and mortality and have a considerable financial impact on the health care system [3, 4]. China has entered an aging society in 1999. According to the Chinese Academy of Social Sciences in 2013, China's elderly population has exceeded 200 million, accounting for about 15.5% of the total population, and will grow at a rate of 1 million per year by 2025 [5]. Xinjiang is located in the northwest border of China, as early as 1995 into the aging society. Curtis [6] pointed out that the impact of gender on bone mass is mainly related to osteoclasts, osteoblasts. Many experiments have shown that estrogen can promote bone growth at the same time [7]. Horiuchi et al. [8] showed that the intake of soybeans was positively correlated with BMD in postmenopausal women. Since there are much more postmenopausal patients than non-postmenopausal patients, the decline in estrogen levels in postmenopausal patients is what causes OP. Rozenberg [9] recommended using menopausal hormone treatment in women who just went through menopause and had minimal baseline risks for breast cancer, thromboembolic disease, cardiovascular disease, and cerebrovascular illness in order to maintain bone health and prevent future fractures. Furthermore, LRP5 is known to act as a protective factor for type-2 diabetes mellitus (T2DM), promoting insulin signaling in addition to increasing insulin production and lowering blood glucose, and the expression of LRP5 is critical for bone formation, especially osteoblasts [10]. It was found that the interaction between LRP5 and LRP6 gene polymorphisms, menopausal age, and blood glucose level in postmenopausal populations could increase the risk of OP. At present, there are no reports on the relationship between the polymorphisms of LRP5 gene rs7125942 and rs3736228 in postmenopausal women and systemic metabolic reduction diseases. Our team's previous research also showed that the expression of polymorphisms of LRP5 gene loci in postmenopausal women is related to BMD abnormalities. Therefore, the aim of this study was to evaluate BMD status and major affecting factors in the middle-aged and aging population in Xinjiang and exploring the relationship between LRP5 gene polymorphisms and mutations and BMD is expected to reveal new biological pathways and lay a foundation for the prevention and treatment of OP.

## Materials and methods

### Research subjects

Participants were conducted from 13 communities in Shihezi, Xinjiang, from May 2022 to May 2023. Diagnostic criteria for type-2 diabetes mellitus (T2DM) and OP are based on the diagnostic criteria established by the WHO in 1999 and 1994, respectively [11, 12].

Patients meeting the following criteria were excluded: (I) gastrointestinal and renal disorders affecting calcium and vitamin D absorption and regulation; (II) severe cardiac disease; (III) liver disease; (IV) malignancy; (V) other congenital osteoporosis; (VI) long-term oral medications affecting bone metabolism.

### Method

#### Collection of baseline data

A total of 136 subjects were measured and recorded for age, height, weight, menopausal, and BMI; Morning fasting venous blood (5 ml) was collected from each subject and serum concentrations of FBG, serum Ca, serum P, serum ALP, cholesterol (TC), triglycerides (TG), creatinine (Cr), blood urea nitrogen (BUN), and uric acid (UA) aminotransferases were measured by using the Roche Automated Biochemistry Analyzer (Modular DPP-H7600). HbAlc was determined by high-pressure liquid chromatography.

#### Measurement of BMD

By dual-energy X-ray absorption (DEXA) measured lumbar spine L1-L4, total L, femoral neck BMD, and Ward triangle (g/cm2), with T values from − 1 to + 1 being normal, − 1 to − 2.5 being osteopenia, and ≤ 2.5 being OP.

#### Gene distribution

LRP5 locus polymorphisms were performed using time-of-flight mass spectrometry.

All of the subjects were provided with comprehensive written informed consent before the study. This study was approved by the Shihezi University Ethics Committee in Xinjiang, China, and strictly adhered to the principles of the Declaration of Helsinki.

### Statistical analysis

Continuous variable data are expressed as mean values (SD). Comparison of mean in multiple groups was conducted with analysis of variance, baseline data were inconsistent using covariance analysis and the χ 2 test was used in the comparison of enumeration data. SPSS 25.0 software was used for statistical analysis. Statistical significance was set at *P* < 0.05.

## Results

### Baseline data of subjects

A total of 136 postmenopausal women were used in our study. According to bone mineral density measured data, 53 people in the group with NBM, 52 patients with osteopenia, and 31 patients with OP. The findings revealed that group C and group B were substantially older than group A (*P* < 0.01). There were no significant differences in BIM and WHR between the three groups, and the baseline data were not the same (Table 1).

**Table 1:** Comparison of baseline information between groups ( x ± s)

Groups	Group A	Group B	Group C	*F*	*P* value
Age (years)	66.08 ± 7.80	69.67 ± 6.47*	71.06 ± 7.06**	5.738	0.04
menopausal (years)	16.23 ± 7.46	19.79 ± 6.07**	21.06 ± 7.06**	5.939	0.03
BMI (kg/m^2^)	26.39 ± 3.59	26.23 ± 4.24	24.76 ± 3.12	2.042	0.13
WHR	0.88 ± 0.13	0.90 ± 0.07	0.90 ± 0.06	0.408	0.67

^*^P < 0.05, **P < 0.01 compared with Group A

### Metabolic characteristics of subjects

**Fig. 1:**
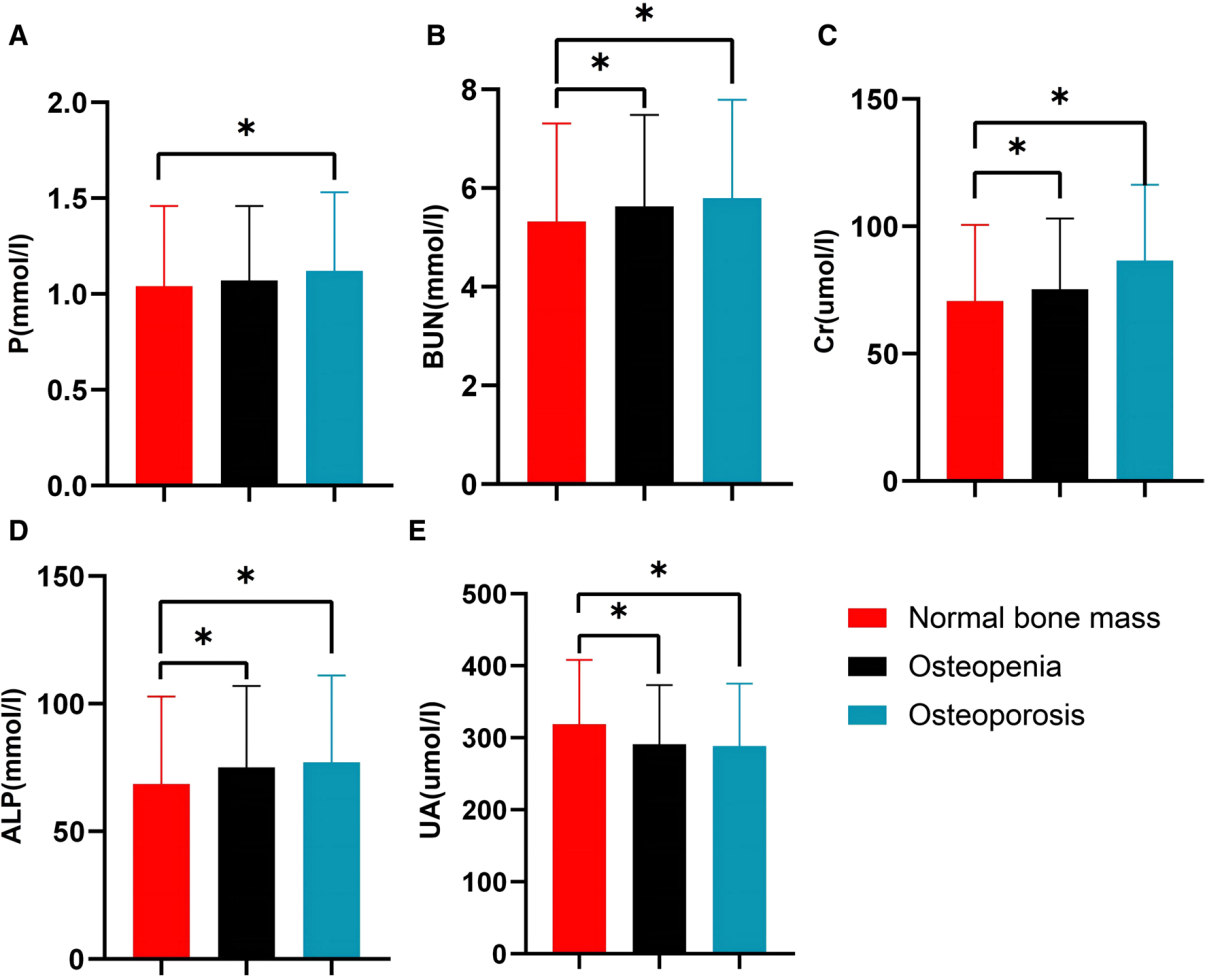
Comparison of biochemical indicator after Covariance analysis (*P < 0.05)

Through the correction of age, serum ALP, Cr, and BUN all showed an upward trend in the three groups, with group C being the highest. On the contrary, serum UA showed a gradual downward trend, with the lowest in the group C. Compared with the group A, the difference in serum ALP, Cr, BUN, and UA between the group B and the group C was statistically significant (*P* < 0.05). In addition, serum P was higher in the group C compared with group A, and the difference was statistically significant (*P* < 0.05) (Fig. 1).

### The relationship between diabetes mellitus and bone abnormalities

There was no difference in diabetes prevalence between the three groups (*P* > 0.01), (Table 2).

**Table 2:** The relationship between menopause and diabetes mellitus and susceptibility to abnormal bone mass

		Group A	Group B	Group C	*χ*^2^	*P*
T2DM	No	25 (18.4)	15 (11.0)	13 (9.6)	3.854	0.146
	Yes	28 (20.6)	37 (27.2)	18 (13.2)		

### Diagnostic value of commonly used sites for bone mineral density

**Fig. 2:**
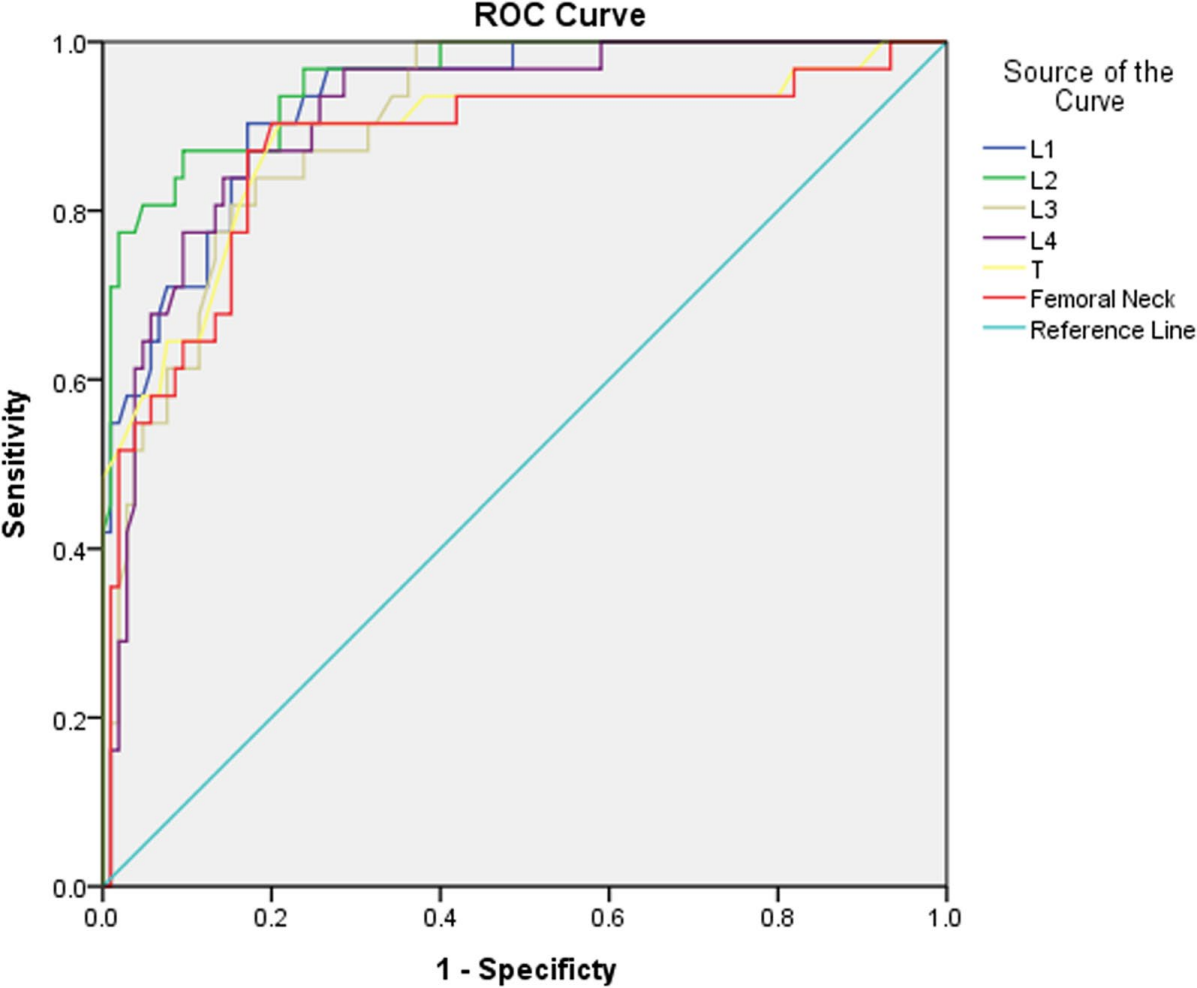
ROC curve for diagnosis of osteoporosis

The receiver operating characteristic (ROC) curve was used to analyze the diagnostic value of L1, L2, L3, L4, total L, and femoral neck for bone mass abnormalities. The total area of L1, L2, L3, L4, total L, the area under the curve of the femoral neck were 0.929, 0.955, 0.901, 0.914, 0.885, and 0.873 (*P* < 0.01). The results showed that the above components were valuable for the diagnosis of OP, and the area under the total L2 was the largest, which had greater clinical diagnostic value (Fig. 2).

### The relationship between genetic mutations and abnormal bone mass

According to the results of BMD measurement, the study subjects were divided into normal bone mass group (NBM) (*n* = 53) and abnormal bone mass abnormal (ABM) group (*n* = 83). The distribution of SNP genotyping at these two loci in LRP5 gene conformed to Hardy–Weinberg's law of genetic equilibrium (*P* > 0. 05). At the rs7125942 locus, there was statistically significant difference in the distribution of wild-type (CC) and mutant (CG) with the NBM group and the ABM group (*P* < 0.05). (Table 3). At rs3736228 locus, there was no significant difference between wild-type (CC) and mutant (CT/TT) in the NBM group and the ABM group (*P* > 0.05).

**Table 3:** Relationship between wild-type, mutant type, and susceptibility to abnormal bone mass at the LRP5 locus

Genotype		Normal bone mass	Abnormal bone mass	χ2	*P*
rs3736228	CC	39 (43.3)	51 (56.7)	2.129	0.144
	CT/TT	14 (30.4)	32 (69.6)		
rs7125942	CC	44 (35.8)	79 (64.2)	5.534	0.019
	CG	9 (69.2)	4 (30.8)		

### Comparison of wild-type and mutant biochemical indexes of LRP5 gene rs3736228 and rs7125942 locus between in the NBM group and the ABM group

At rs7125942 locus, compared with wild-type (CC), mutant (CG) had lower FPG and LDL (*P* < 0.05) in NBM group, and lower serum ALP in the ABM group (*P* < 0.05). At rs3736228 locus, compared with wild-type (CC), the BMD (Femoral neck) of mutant-type (CT/TT) was lower in the NBM group (*P* < 0.05) (Tables 4, 5).

**Table 4:** Comparison of wild-type and mutant biochemical indexes of LRP5 gene rs7125942 locus between normal bone mass group and abnormal bone mass group

Variables	Normal bone mass		Abnormal bone mass	
	CC	CG	CC	CG
FPG (mmol/L)	6.78 ± 3.20	5.27 ± 0.85*	6.65 ± 1.95	6.39 ± 1.11
HbA1C%	6.68 ± 1.38	6.16 ± 0.62	6.93 ± 1.18	7.78 ± 1.43
TG (mmol/L)	2.46 ± 2.11	1.99 ± 1.63	1.65 ± 1.00	1.41 ± 0.43
LDL (mmol/L)	3.31 ± 1.04	2.41 ± 0.52*	3.32 ± 0.98	3.84 ± 0.75
HDL (mmol/L)	1.26 ± 0.38	1.26 ± 0.22	1.25 ± 0.30	1.26 ± 0.46
Ca (mmol/L)	2.26 ± 0.20	2.29 ± 0.08	2.28 ± 0.10	2.27 ± 0.05
P (mmol/L)	1.10 ± 0.10	1.07 ± 0.11	1.20 ± 1.00	1.12 ± 0.15
ALP (mmol/L)	76.52 ± 18.83	77.78 ± 18.43	76.58 ± 22.30	70.50 ± 3.32*
BMD (L1–L4)	1.21 ± 0.15	1.18 ± 0.15	0.88 ± 0.14	0.97 ± 0.11
BMD (Femoral neck)	0.85 ± 0.29	0.87 ± 0.13	0.73 ± 0.11	0.71 ± 0.10

^*^P < 0.05 compared with CC genotype

**Table 5:** Comparison of wild-type and mutant biochemical indexes of LRP5 gene rs3736228 locus between normal bone mass group and abnormal bone mass group

Variables	Normal bone mass		Abnormal bone mass	
	CC	CT/TT	CC	CT/TT
FPG (mmol/L)	6.70 ± 3.18	6.03 ± 2.34	6.77 ± 2.06	6.42 ± 1.65
HbA1C%	6.67 ± 1.36	6.36 ± 1.08	7.09 ± 1.34	6.78 ± 0.92
TG (mmol/L)	2.40 ± 1.94	2.33 ± 2.33	1.67 ± 0.99	1.58 ± 0.99
LDL (mmol/L)	3.23 ± 0.81	3.11 ± 0.81	3.42 ± 0.99	3.23 ± 0.95
HDL (mmol/L)	1.21 ± 0.35	1.40 ± 0.35	1.25 ± 0.32	1.24 ± 0.28
Ca (mmol/L)	2.26 ± 0.22	2.26 ± 0.06	2.27 ± 0.10	2.29 ± 0.09
P (mmol/L)	1.07 ± 0.10	1.14 ± 0.11	1.28 ± 1.25	1.05 ± 0.12
ALP (mmol/L)	78.95 ± 19.25	70.57 ± 14.59	76.47 ± 21.11	76.00 ± 24.79
BMD (L1–L4)	1.23 ± 0.16	1.13 ± 0.09	0.89 ± 0.15	0.89 ± 0.11
BMD (Femoral neck)	0.90 ± 0.11	0.72 ± 0.47*	0.74 ± 0.11	0.73 ± 0.12

^*^P < 0.05 compared with CC genotype

The variables showing negative correlation of femoral neck BMD are age and menopausal years (*P* < 0.01), and L1-4 bone density is negatively correlated variables are age, menopausal years (*P* < 0.01), and the variables that show positive correlation are BMI and TG (*P* < 0.05) (Table 6).

**Table 6:** Pearson correlation analysis of bone density between femoral neck and L1-4 for each variable

Variables	BMD (Femoral neck)		BMD (L1-4)	
	r	*P*	r	P
Age (years)	− 0.263	0.002	− 0.245	0.004
Years of menopause (years)	− 0.269	0.002	− 0.249	0.003
BMI (kg/cm^2^)	0.161	0.061	0.194	0.023
WHR	0.094	0.277	0.126	0.143
FPG (mmol/L)	0.051	0.553	0.082	0.344
HbA1C%	− 0.003	0.974	0.042	0.632
TG (mmol/L)	0.139	0.105	0.276	0.001
HDL (mmol/L)	− 0.009	0.254	− 0.065	0.450
LDL (mmol/L)	0.097	0.261	− 0.037	0.665
Ca (mmol/L)	− 0.006	0.941	− 0.033	0.702
P (mmol/L)	− 0.085	0.326	− 0.059	0.494
rs556442	0.025	0.771	− 0.009	0.918
rs312778	− 0.072	0.394	0.075	0.375
rs4988331	0.060	0.489	0.127	0.142
rs3736228	0.149	0.084	− 0.024	0.783

The statistically significant variables in Table 6 were included in the multiple linear regression analysis, and were not included in the model at the same time due to the collinear relationship between age and menopausal years, and age was included here. Taking L1-4 and femoral neck BMD as the dependent variables, the results showed that age, BMI, and TG could enter the multivariate logistic regression model of BMD (L1-4) (*P* < 0.05), and the regression equation was *Y*(L1-4) = 1.154–0.007*age + 0.010*BMI + 0.033*TG. Age could enter the multivariate logistic regression model of femoral neck BMD (*P* < 0.05), and the regression equation was *Y*(femoral neck) = 1.254–0.007*age (Table 7).

**Table 7:** BMD multiple linear regression and analysis

		β	SE	P
BMD (Femoral neck)	Constant	1.254	0.151	0.000
	Age (years)	− 0.007	0.002	0.002
BMD (L1-4)	Constant	1.154	0.196	0.000
	Age (years)	− 0.007	0.002	0.005
	BMI (kg/cm^2^)	0.010	0.005	0.035
	TG (mmol/L)	0.033	0.011	0.004

## Discussion

OP is a common disease in middle-aged and elderly patients, and the most serious complication is the occurrence of fracture. Shihezi, Xinjiang is a city of army reclamation, it is located in the northwestern border of China and entered the aging society as early as 1995 [13]. OP has grown to be a significant public health issue. The relationship between gene polymorphisms and mutations in LRP5, the classical receptor of WNT signaling pathway, and glucose, lipid metabolism, and bone mass reduction has also become a research hotspot in recent years.

Our study showed that increasing age is a risk factor for osteopenia and osteoporosis, Clinical Update on Osteoporosis concluded that the key factors affecting OP fractures included a low body mass index (20 kg/m2 or 127 pounds), current smoking, a history of osteoporotic fractures in the family, early menopause (age of < 40 years old) and other factors [14]. At present, numerous investigations have demonstrated that one of the persistent consequences of DM is OP [15–17]. However, our study showed that BMI and the presence or absence of diabetes were not significantly related to bone mass abnormalities, which may be related to sample selection.

Hutchison et al. [18] found that 50% of the patients had histological changes in the bone when half of the normal glomerular filtration rate was achieved. Our study confirmed that renal function indicators of serum Cr, serum BUN in the OP group, osteopenia group, and controls group decreased, respectively. A cross-sectional study of older men found that BMD levels in the normal high values of serum UA were higher than that in the normal low values [19]. Studies have demonstrated that UA can result in intracellular mitochondrial malfunction, which lowers the generation of adenosine triphosphate and negatively affects endothelial function, thereby affecting blood supply to bone tissue and significantly reducing bone turnover [20]. Our findings demonstrated that UA levels were successively increased in the OP, osteoporosis and control groups, suggesting that controlling UA levels to normal high values could reduce or delay the development of OP. Our study showed that higher serum ALP was associated with lower femoral BMD, which was similar to Filippo Migliorini’s [21].

The results of our study found that there was statistically significant differences in the distribution of wild-type (CC) and mutant (CG) groups with normal bone mass and abnormal bone mass groups in rs7125942 locus (*P* < 0.05). It was suggested that gene mutations in rs7125942 may increase the risk of OP. At rs7125942 locus, compared with wild-type (CC), mutant (CG) had lower FPG and LDL in the normal bone mass group, and lower serum ALP in the bone abnormality group. At rs3736228 locus, the BMD (Femoral neck) of mutant-type (CT/TT) was lower  than that of wild-type (CC) in the normal bone mass group (*P* < 0.05). The results of multiple linear regression analysis showed that BMI was positively correlated with BMD of femoral neck and lumbar L1-4, and the increase of BMI within a certain range was of positive significance for delaying OP progression, which was consistent with the results of Fan [22], but this was contrary to the conclusion of Wang et al. [23], indicating that the increase of BMI beyond a certain range may be used as a risk factor for OP. Decreased TG levels are positive for delaying bone loss, and this conclusion is that, in contrast to Aleidi et al. [24], increased age and menopausal years are risk factors for OP.

In summary, Xinjiang area is a high prevalence area of OP, and especially in postmenopausal women. Mutations in the LRP5 gene rs7125942 may be related to bone metabolism in postmenopausal women. In this study, the influencing factors of bone metabolism disorders in postmenopausal women in Shihezi area of Xinjiang were explored from the genetic level. The limitation of this study is that we only selected postmenopausal women as the research subjects, and its findings may have limited generalizability beyond the population of postmenopausal women, and bone density may be affected by racial genetic differences, diet, and region. In addition, it is necessary to expand the population sample to explore the effects of gender and sex hormones on bone metabolism, and treatments and interventions for osteopenia need to be further studied. This study provides a basis for gene targeted prevention and treatment of OP.

## Conclusion

Meanwhile, the effects of serum ALP, TG, BUN, Cr, and P levels and abnormal renal function on BMD should be emphasized; measurement of L2 BMD is more meaningful in the diagnosis of OP in Xinjiang, China. LRP5 gene rs7125942 site mutation is related to BMD.

## Data Availability

All data, models, or code used during the study are available from the corresponding author by request.
